# Dietary Regulation of Gut-Brain Axis in Alzheimer’s Disease: Importance of Microbiota Metabolites

**DOI:** 10.3389/fnins.2021.736814

**Published:** 2021-11-19

**Authors:** Dulce M. Frausto, Christopher B. Forsyth, Ali Keshavarzian, Robin M. Voigt

**Affiliations:** ^1^Rush Medical College, Rush Center for Integrated Microbiome and Chronobiology Research, Rush University Medical Center, Chicago, IL, United States; ^2^Department of Medicine, Rush University Medical Center, Chicago, IL, United States; ^3^Department of Physiology, Rush University Medical Center, Chicago, IL, United States

**Keywords:** Alzheimer’s disease, microbiota, diet, SCFA, LPS, bacterial metabolites, intestinal hyperpermeability

## Abstract

Alzheimer’s disease (AD) is a neurodegenerative disease that impacts 45 million people worldwide and is ranked as the 6th top cause of death among all adults by the Centers for Disease Control and Prevention. While genetics is an important risk factor for the development of AD, environment and lifestyle are also contributing risk factors. One such environmental factor is diet, which has emerged as a key influencer of AD development/progression as well as cognition. Diets containing large quantities of saturated/trans-fats, refined carbohydrates, limited intake of fiber, and alcohol are associated with cognitive dysfunction while conversely diets low in saturated/trans-fats (i.e., bad fats), high mono/polyunsaturated fats (i.e., good fats), high in fiber and polyphenols are associated with better cognitive function and memory in both humans and animal models. Mechanistically, this could be the direct consequence of dietary components (lipids, vitamins, polyphenols) on the brain, but other mechanisms are also likely to be important. Diet is considered to be the single greatest factor influencing the intestinal microbiome. Diet robustly influences the types and function of micro-organisms (called microbiota) that reside in the gastrointestinal tract. Availability of different types of nutrients (from the diet) will favor or disfavor the abundance and function of certain groups of microbiota. Microbiota are highly metabolically active and produce many metabolites and other factors that can affect the brain including cognition and the development and clinical progression of AD. This review summarizes data to support a model in which microbiota metabolites influence brain function and AD.

## Epidemiology of Dementia and Alzheimer’s Disease

Dementia currently effects more than 50 million people worldwide and that number is predicted to increase as the population ages, reaching 75 million in 2030 and 131.5 million by 2050 (2019 ALZHEIMER’S Disease Facts And Figures, 2019). 2019 ALZHEIMER’S DISEASE FACTS AND FIGURES Includes a Special Report on Alzheimer’s Detection in the Primary Care Setting: Connecting Patients and Physicians; (Alzheimer’s Association, 2020; Longhe, 2020). On the Front Lines: Primary Care Physicians and Alzheimer’s Care in America), Dementia is characterized as a chronic or progressive deterioration in cognitive function, beyond the expectations of normal aging, sufficient to cause dependence, disability and mortality. Reports predict that the largest increase in dementia prevalence will be in low and middle class countries, which currently have increasing prevalence of cardiovascular disease, hypertension and diabetes [risk factors for Alzheimer’s disease (AD)] (Faraco and Iadecola, 2013; Prince et al., 2014; Thorin, 2015). In the past decade, risk factors for vascular disease have been associated with many types of dementia including AD (Duron and Hanon, 2008). Alzheimer’s disease accounts for 50-75% of dementia cases, being the single greatest cause of dementia worldwide (Prince et al., 2014). Alzheimer’s disease is a common disease, affecting millions of people across the world and approximately 5.7 million Americans (2019 ALZHEIMER’S Disease Facts And Figures, 2019). 2019 ALZHEIMER’S DISEASE FACTS AND FIGURES Includes a Special Report on Alzheimer’s Detection in the Primary Care Setting: Connecting Patients and Physicians; On the Front Lines: Primary Care Physicians and Alzheimer’s Care in America. In 2019, AD was the 6th leading cause of death in the United States with annual costs of care exceeding $200 billion (2019 ALZHEIMER’S Disease Facts And Figures, 2019). 2019 ALZHEIMER’S DISEASE FACTS AND FIGURES Includes a Special Report on Alzheimer’s Detection in the Primary Care Setting: Connecting Patients and Physicians.

Alzheimer’s disease is a devastating neurodegenerative disease clinically defined by progressive debilitating, multi-domain cognitive impairment that is distinct from that observed during normal aging (McKhann et al., 1984; Teri et al., 1997). Alzheimer’s disease includes multi-faceted cognitive impairments that interfere with day-to-day functioning, including memory, thinking, judgment, language, problem-solving, personality, and movement (Carlesimo and Oscar-Berman, 1992; Panegyres, 2004). This decline is due to neuronal dysfunction and death in parts of the brain involved in cognitive function. Eventually, AD affects other parts of the brain and AD patients exhibit psychological symptoms (Ropacki and Jeste, 2005; Shimabukuro et al., 2005), visuospatial deterioration (Cronin-Golomb et al., 1995), and extreme motor dysfunction that worsens during the late and terminal stages of AD (Suvà et al., 1999). People in the final stages of the disease are bed-bound and require around-the-clock care. Ultimately, the AD prognosis is fatal. Although AD shortens an individual’s life span, it is usually not the direct cause of death (Brunnström and Englund, 2009). Rather, individuals diagnosed with AD experience significant health complications. Prevalent co-morbid conditions include: bedsores, undiagnosed urinary tract infections, general infections, sepsis, injuries from falls, malnutrition and dehydration (Longhe, 2020). Overall, AD develops slowly and gradually worsens over the span of several years (if not decades), eventually affecting most areas of the brain.

Alzheimer’s disease has been recognized by the World Health Organization (WHO) as a global public health priority because of high incidence but also because there is a lack of effective treatments to modify disease progression or significantly ameliorate symptoms (Cummings et al., 2014, 2018). In fact, treatments for AD remain elusive. AD has the highest failure rate in clinical treatment trials of any disease, which is evident by a 99.6% failure rate and with only a handful of drugs approved for AD treatment out of hundreds of trials (Cummings et al., 2014, 2018; Hay et al., 2014). For the most part, treatments for AD focus on stabilizing neurotransmitters, such as increasing acetylcholine and decreasing glutamate uptake, with the goal of facilitating connections between neurons by promoting synaptic plasticity and neuronal survival. Additionally, other treatments may prevent some of the pathological hallmarks of AD including amyloid-beta (Aβ) plaque and neurofibrillary tangle formation, but these treatments are often associated with toxicity and appear to have limited clinical utility. Studies have found that AD treatments targeted toward reducing Aβ only mildly ameliorate symptoms. For example, in 2021 the United States Food and Drug Administration (FDA) approved the first AD treatment since 2003. Although, this treatment, Aduhelm (aducanumab), consistently reduces the level of amyloid plaques in the brain, it failed to produce a meaningful clinical impact (Alexander and Karlawish, 2021). This lack of clinical impact reinforces the necessity for the development of new, innovative mechanistic AD disease models and treatments.

The increased prevalence of AD in conjunction with the lack of effective treatment presents us with an urgent health care crisis. Innovative treatment approaches are urgently needed but this cannot occur until we develop a better understanding of the pathological process leading to AD development and progression. Perhaps, treatments are not successful due to the incorrect AD models currently available.

## Pathology of Alzheimer’s Disease

Neuropathologically, AD is characterized by Aβ protein plaques and hyper-phosphorylation of tau protein (Serrano-Pozo et al., 2011). In fact, clinical diagnosis of AD is based on decline in cognition and positive biomarker assays of Aβ and/or tau in positron emission tomography (PET) scans and in cerebral spinal fluid (CSF) levels, or in post-mortem brain tissue (Jack et al., 2011; Blennow et al., 2015; Knopman et al., 2016; Selkoe and Hardy, 2016).

### Amyloid-Beta Plaques

**FIGURE 1:**
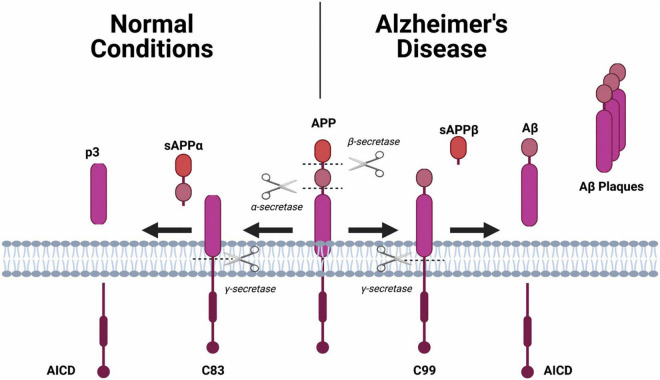
The amyloid precursor protein (APP) will undergo one of two different hydrolysis pathways. **Normal Condition:** Sequential cleavage of APP by α-secretase and γ-secretase generates a soluble amino terminal ectodomain of APP (sAPPα), the carboxy terminal fragment C83, APP intracellular domain (AICD) and a short fragment p3. **Alzheimer’s Disease:** Sequential cleavage of APP by β-secretase (BACE1) and γ-secretase generates sAPPβ, C99, AICD and Aβ.

Amyloid-beta precursor protein (APP), is found in many tissues and organs, including the brain and spinal cord and is critical for the normal function of neurons (Drummond and Wisniewski, 2017). Under normal conditions, APP is cleaved by two enzymes, α-secretase and γ-secretase, which results in the generation of soluble products (i.e., p3, sAPPα, AICD) (Figure 1; Drummond and Wisniewski, 2017). However, cleavage of APP by β-secretase and γ-secretase results in a cleavage product that is insoluble, a fragment known as an Aβ monomer (Drummond and Wisniewski, 2017). When there is an accumulation of Aβ monomers, they can cluster and form extracellular plaques around the neurons (Drummond and Wisniewski, 2017). It is thought that these plaques disrupt neuronal health and signaling resulting in brain dysfunction which manifests as symptoms such as impaired memory.

### Neurofibrillary Tangles

**FIGURE 2:**
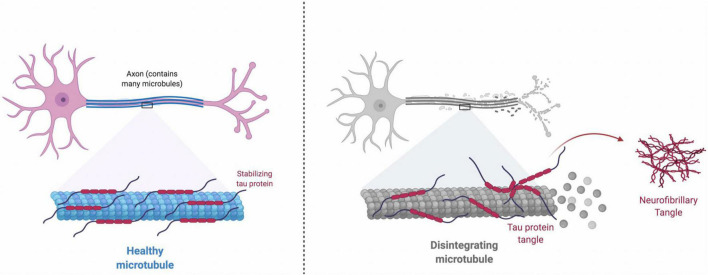
**Healthy Microtubule:** Tau protein binds and stabilizes microtubules. The attachment of tau to microtubules is regulated through a balanced phosphorylation by kinases (e.g., Cdk5, GSK3β, MARK and ERK2) and dephosphorylation by phosphatases (e.g., PP1, PP2A, PP2B, and PP2C). **Disintegrating Microtubule:** Under pathological conditions, equilibrium between the roles of kinases and phosphatases is disrupted, and increase in the kinase activity and decrease in the phosphatase activity will cause tau hyperphosphorylation. Hyperphosphorylated tau protein is misfolded and leads to more organized aggregates, which eventually develops neurofibrillary tangles inside neurons. Neurofibrillary tangles impair normal axonal transport, disrupt synaptic plasticity, and induce cell death.

Neurons, like other cells, have a cytoskeleton which is partly made up of microtubules (Figure 2). Neurofibrillary tangles occur when tau, a protein that keeps microtubules stable and intact, is hyper-phosphorylated (Drummond and Wisniewski, 2017). Once this occurs, microtubule stability is hindered and tau clumps together, forming twisted/tangled fibers inside the neuron (Drummond and Wisniewski, 2017). Neurons with tangles and non-functioning microtubules cannot maintain the cytoskeleton, which results in neuronal death by apoptosis.

### Neuroinflammation

The presence of neuroinflammation is a cardinal feature and thought to be a key driver of AD. Neuroinflammation is the consequence of peripheral immune cells (adaptive and innate) as well as the activation of resident immune cells of the central nervous system (CNS). Perhaps one of the most well studied immune cell in neuroinflammation, including AD, are microglia which are resident immune cells in the brain. Microglia become activated in response to brain trauma, infections and pathogens, and the presence of misfolded proteins (including Aβ and tau plaques) (Milatovic et al., 2017). Microglial activation is characterized by release of inflammatory mediators, such as cytokines and chemokines, and generation of reactive oxygen and nitrogen species (Lucas et al., 2006; Liddelow et al., 2017; Milatovic et al., 2017). Generally, these responses are beneficial and critical for optimal brain function and repair; however, uncontrolled and/or prolonged activation can lead to cell damage and dysfunction which may result in neurodegeneration (Lucas et al., 2006; Tahara et al., 2006; Gambuzza et al., 2014; Liddelow et al., 2017). Indeed, increased markers of microglial activation, such as major histocompatibility complex II (MHCII) and CD68, are noted in AD-relevant brain regions such as the entorhinal cortex, frontal and temporal gyri, hippocampus, and frontal, temporal and occipital cortices in post-mortem AD brain tissue (Gomez-Nicola and Boche, 2015; Hopperton et al., 2017). Additionally, patients with AD demonstrate higher CSF levels of proinflammatory cytokines, such as eotaxin, interleukin (IL)-1ra, IL-1β, IL-4, IL-7, IL-9, IL-10, IL-13, and granulocyte colony-stimulating factor, compared to non-demented controls (Italiani et al., 2018; Taipa et al., 2019).

These pathological hallmarks are well established. However, whether these pathological hallmarks are a cause, or a consequence of the disease is highly debated.

## Alzheimer’s Disease Pathogenesis and Importance of Diet

Genetics and family history are important determinants of AD risk and yet these factors alone only account for approximately 2% of AD cases (Nelson et al., 2011; National Institutes of Health, 2015). This means other factors, such as environment and lifestyle, must also be involved. This is important because it means that modification of environment and lifestyle may be a viable approach to prevent, delay the onset, or modify clinical progression of AD. Factors such as social engagement, psychological and biological stress, physical activity, and diet are all well-established factors that can influence AD development. Of these risk factors, there is compelling evidence that diet is critically important for neuroinflammation and cognition, and will be the focus of this review (Wu et al., 2003; Qin et al., 2006; Subash et al., 2015; Matt et al., 2018; Park et al., 2020).

Literature regarding the impact of individual nutrients or food items on AD risk is complex, partly because humans eat meals with intricate combinations of nutrients that are likely to be synergistic, thereby creating complications when determining which of the dietary components (macronutrients and other nutrients) are exerting an adverse or protective effect. Below is a discussion of macronutrients, other dietary factors, and dietary patterns on neuroinflammation, brain pathology, cognitive function, and risk of AD.

### Macronutrient: Fats

Dietary fat can be categorized as saturated or unsaturated. Unsaturated fats are further divided into monounsaturated fatty acids (MUFA) and polyunsaturated fatty acids (PUFA, including omega-3 fatty acids such as docosahexaenoic acid (DHA) and eicosapentaenoic acid). Trans-fats are partially hydrogenated unsaturated fats that extend the shelf life of foods. Saturated and trans-fats (“bad fats”) are generally considered to have adverse effects on human health including associations with AD, conversely MUFA and PUFA (“good fats”) are thought to have beneficial effects, including improved brain function and prevention of neurodegenerative diseases (Field et al., 2001; Ludwig et al., 2018).

Studies in animals have produced evidence to support the negative effects of saturated and trans-fat on AD-like pathology and behavior. For example, consumption of a diet composed of high levels of saturated and trans-fat is sufficient to enhance cerebrovascular Aβ deposition, hippocampal oxidative stress, and induce cognitive impairment in a mouse model of AD (5xFAD) (Lin et al., 2016). Additionally, intake of a diet composed of 42% fat (vs. control, which contains 13% fat) is associated with impaired memory, an increase in Aβ monomers and plaques, and brain inflammation in an AD transgenic mouse model (APP/PS1) (Bracko et al., 2020). Indeed, these neuropathological changes may have an important cognitive impact. Epidemiological studies show that dietary saturated and trans-fat intake is associated with AD and dementia risk (Luchsinger et al., 2002; Morris et al., 2003; Laitinen et al., 2006). For example, in the Chicago Healthy Aging Project (CHAP), clinical evaluations were performed at two different time points, in aged individuals, and revealed positive associations between both saturated and trans-fat intake and risk of developing AD (Morris et al., 2003). Additional studies in New York and Finland have also generated data to support an increased risk for developing dementia with increased consumption of saturated fat (Luchsinger et al., 2002; Laitinen et al., 2006). Furthermore, a recently conducted meta-analysis systematically examined four independent prospective cohort studies and found that a high intake of saturated fat is significantly associated with an increased risk of AD (39%) and dementia (105%), respectively (Ruan et al., 2018).

On the contrary, studies suggest a protective role for PUFA against age-associated cognitive decline and AD (Wood et al., 2021). A study in rodents demonstrates that consumption of DHA attenuates neuronal loss, and restores neurogenesis in an AD mouse model (5xFAD) (Park et al., 2020). While generally supportive of omega-3 fatty acids being beneficial for cognition, studies looking at fish consumption (an omega-3 fatty acid containing food) have had mixed results, which could perhaps be accounted for by different levels of omega-3 fatty acids found in fish in different parts of the world (Lawson and Hughes, 1988; Morris et al., 2003, 2015b; Huang et al., 2005; Freund-Levi et al., 2006; Schaefer et al., 2006; Nurk et al., 2007; Devore et al., 2009; Li et al., 2011; Rest et al., 2016). Given the differences in omega-3 fatty acids in fish, more insight is gleamed from trials in which a known quantity of omega-3 fatty acids are consumed daily. Daily supplementation of omega-3 fatty acids for 6 months in the OmegAD trial improved cognitive performance in individuals with mild AD (although effects in cognitively normal individuals were less apparent) (Eriksdotter et al., 2015). Indeed, similar results are found in other studies in which dietary supplementation of omega-3 fatty acids improved cognition with the greatest effects observed in those with mild cognitive impairment or at the low end of cognitively normal (Kotani et al., 2006; Lee et al., 2013; Mahmoudi et al., 2014; Hamel et al., 2015; Andrieu et al., 2017; Soininen et al., 2017; Zhang et al., 2018). Moreover, improvements following DHA supplementation may be maintained for a period beyond the DHA treatment (Quinn et al., 2010; Yurko-Mauro et al., 2010; Zhang et al., 2016). Interestingly, this beneficial effect has not been observed in all studies including the Multidomain Alzheimer Preventive Trial (MAPT) and Alpha Omega Trial, however this may be a reflection of different doses or other factors associated with subject populations (Scheltens et al., 2010; Geleijnse et al., 2012; Phillips et al., 2015; Andrieu et al., 2017; Tabue-Teguo et al., 2018).

Taken together, diets low in saturated/trans-fats (i.e., bad fats), high in mono/polyunsaturated fats (i.e., good fats) are associated with reduced risk of AD and slower age-associated cognitive decline (Scarmeas et al., 2006, 2007; Tangney et al., 2011; Gardener et al., 2012; Matura et al., 2021).

### Macronutrient: Carbohydrates

Carbohydrates can be categorized into different groups which are broadly separated into simple sugars (monosaccharides, disaccharides) and complex carbohydrates (starch, fiber). Additionally, refined carbohydrates are those that have been highly processed and often have added sugars and sweeteners (e.g., sucrose, fructose, high fructose corn syrup). Diets high in simple sugars and refined carbohydrates (e.g., white rice, white bread), are associated with increased risk of poor cognitive performance, dementia, and AD; conversely diets high in complex carbohydrate fiber are associated with decreased risk of AD. For example, a clinical trial found that a high-sugar, high-fat diet is associated with impaired memory which was not observed in individuals consuming a diet that contained less sugar and fat (Attuquayefio et al., 2017). However, as already discussed, the dietary fat that was included in this trial could have contributed to this effect. Nonetheless, consumption of a high-carbohydrate diet is associated with cognitive dysfunction in people of advanced age (Roberts et al., 2012).

Indeed, human studies demonstrate detrimental effects of refined carbohydrate intake (Pistollato et al., 2018; Gentreau et al., 2020; Miao et al., 2020). Studies report that total sugar intake is inversely associated with cognitive function (Ye et al., 2011; Chong et al., 2019). Specifically, data from the Framingham Heart Study show that consumption of soft drinks (and fruit juice) are associated with dose-dependent reductions in hippocampal volume and poor memory (Pase et al., 2017; Handing et al., 2019). Similarly, in 2020, a 20-year prospective cohort study reported an association between long-term sugar consumption from beverages (i.e., soft drinks) and dementia, including AD (Miao et al., 2020). Studies in animal models provide mechanistic evidence of why this association may occur. Consumption of high amounts of fructose is sufficient to induce neuroinflammation and impair neurogenesis in several animal models (van der Borght et al., 2011; Frias et al., 2014; Yin et al., 2014; Hsu et al., 2015; Li et al., 2015; Xu et al., 2016; Cigliano et al., 2018; Jiménez-Maldonado et al., 2018). Furthermore, fructose consumption promotes learning and memory deficits as well as Aβ deposition in the hippocampus and cortex of rats (Stranahan et al., 2008; Ross et al., 2009; Luo et al., 2011; Wu et al., 2015; Sangüesa et al., 2018). Administration of simple sugars to AD mouse models also provides important insight about how sugar may impact cognition and risk of dementia, and AD. For example, high levels of sucrose intake in a transgenic AD mouse model (3xTg) promotes neuronal dysfunction (impaired hippocampal neurogenesis and synaptic plasticity), as well as memory impairment (Ferreiro et al., 2020). Similarly, high intake of refined carbohydrates increases memory dysfunction and insoluble Aβ protein levels in the brain of an amyloidosis transgenic mouse model of AD (APP/PS1) (Lin et al., 2016). Overall, these mechanisms could underline the observations made in humans, however, additional studies are needed to confirm this assertion.

Unlike simple sugars and refined carbohydrates, dietary fiber, the portion of plant-derived food that cannot be digested by mammalian enzymes, has been found to be associated with beneficial effects on memory and brain health, in both animal models and human studies (Franco et al., 2005; Smith and Wilds, 2009; Nelson et al., 2012; Martins and Fernando, 2014; Collins and Reid, 2016; Matt et al., 2018; Sindi et al., 2018; Desmedt et al., 2019; Hoffman J.D. et al., 2019; Swann et al., 2020). Human studies have examined the impact of dietary patterns which are characterized by high intake of fiber-containing foods such as the Mediterranean Diet, Dietary Approaches to Stop Hypertension (DASH), and the MIND Diet, which consistently demonstrate decreased risk of AD (Martins and Fernando, 2014; Richard et al., 2018; van den Brink et al., 2019). Needless to say, these are complex diets which make it difficult to isolate the effects of carbohydrates compared to other dietary components like fat and protein. Nevertheless, a number of human trials have examined the effects of different dietary fibers and found evidence to support the notion that consumption of fiber is beneficial for brain health (Best et al., 2009, 2015; Smith et al., 2015). For example, short-term consumption of prebiotic supplements (i.e., oligofructose-enriched inulin) are sufficient to improve short-term memory in healthy individuals (Smith et al., 2015). Likewise, further studies demonstrate beneficial effects of a single-dose of prebiotic supplementation (i.e., non-starch polysaccharide) on cognitive performance in healthy middle-aged adults, characterized by enhanced memory performance and indicators of well-being (Best et al., 2009, 2015). Rodent studies largely recapitulate these findings and suggest that dietary fiber may induce these encouraging effects by targeting microglia and neuroinflammation (Messaoudi et al., 2005; Han et al., 2010; Waworuntu et al., 2014). For example, consumption of a high-fiber diet (5% inulin) in aged mice attenuates pro-inflammatory gene expression in microglia (Matt et al., 2018). Additionally, the fiber inulin reduces inflammatory gene expression in the hippocampus of an aged transgenic AD mouse model (*APOE4*) (Hoffman J.D. et al., 2019).

Additional studies are needed to fully understand the relationship between fiber and cognitive function but the data so far suggest these types of studies are warranted.

### Macronutrient: Proteins

Protein is an essential macronutrient that is found in every cell of the human body, which is used to build and maintain bone, muscle and skin. Dietary protein is obtained from consumption of meat, dairy products, nuts, and certain grains and beans. Although, protein is essential for the human body, the amounts and types of protein (e.g., animal-derived protein, plant-based protein) consumed can have varying effects on the health and function of the human brain. Data recently published from the Nurse’s Health Study demonstrates that long-term protein consumption may influence risk of developing cognitive decline wherein higher protein is associated with less subjective cognitive impairment (Yeh et al., 2021). This may not be surprising since studies have demonstrated that as many as 50% of adults of advanced age do not eat the recommended daily amount of protein (National Center for Health Statistics, 2019).

Moreover, the type of protein consumed may also be critical. Generally, consumption of high amounts of animal-derived protein have detrimental effects on cognitive function, while plant-based protein has protective effects on brain health (Albanese et al., 2009; Grant, 2014; Sun et al., 2021; Zhang et al., 2021). Indeed, a recent cohort study identified high-meat consumption as a potential risk factor for incidence of dementia (Zhang et al., 2021). Accordingly, a study conducted in Sweden on cognitively healthy individuals showed that an overall decrease in dietary meat and meat product intake is associated with better cognitive performance in clinical dementia screening tests, thus underlining the negative effects high-meat intake can have on brain health (Titova et al., 2013). Previous reviews, have also found a pattern between high meat and meat product intake with increased AD prevalence, while substitution with plant-based protein is associated with lower risk of dementia-related mortality and improved brain health (Martins and Fernando, 2014; van den Brink et al., 2019). Although, evidence does support a link between protein consumption and brain health, it is important to note, protein consumption is typically embedded as part of complex dietary patterns with considerable heterogeneity, and other macronutrients aside from protein, such as fat and carbohydrates may play a role. Thus, the evidence linking risk of dementia with specific type or amount of protein consumption is limited.

Although, there is a lack of understanding of the relationship between protein intake and direct effects on cognitive function and dementia, there is evidence to support a link between protein and neuroinflammation, which is a critical mechanism responsible for determining brain health (Barbaresko et al., 2013; Sreeja et al., 2014; Snelson et al., 2017). Consumption of high amounts of animal-derived protein are associated with a pro-inflammatory immune profile, while intake of plant-based protein has been tied to anti-inflammatory effects (Barbaresko et al., 2013; Sreeja et al., 2014; Snelson et al., 2017). In 2013, a comprehensive meta-analysis (46 epidemiological studies) investigated associations between dietary patterns and biomarkers of inflammation in humans, and found a positive association between animal-based diets and levels of inflammatory biomarkers, while plant-based diets showed no association (Barbaresko et al., 2013). Accordingly, animal studies show that consumption of diets enriched in casein (animal protein) can induce immune cell activation and inflammatory cytokine activity in the brain, while supplemented intake of plant-based soy protein protects the brain from oxidative damage and inflamamtion (Sreeja et al., 2014; Snelson et al., 2017). Accordingly, a recent publication using the senescence-accelerated mouse-prone 8 (SAMP8) mice (a model for aging and dementia), found that consumption of animal-derived protein is enough to promote gliosis, neuroinflammation, and impair memory when compared to consumption of a soy-protein diet (Petralla et al., 2020).

Overall, further studies are necessary to adequately isolate the effects of different protein types to cognitive impairment. However, there is enough data to suggest a potential link between negative effects of animal-derived protein and protective effects of plant-based protein on cognitive function and brain health.

### Other Dietary Components: Polyphenols

Polyphenols are micronutrients with anti-oxidant properties that naturally occur in certain plant-based foods (Pérez-Jiménez et al., 2010). Notably, polyphenols have become popular in the field of aging due to their anti-inflammatory activity *in vitro* and in animal studies, and have been extensively reviewed as potential therapeutic agents for neurodegenerative disease such as AD (Choi et al., 2020). Indeed, *in vitro* studies show that natural polyphenols, such as curcumin, epigallocatechin gallate, and grape seed extract, have the ability to attenuate Aβ aggregation by preventing the formation of toxic amyloid fibrils and convert previously existing amyloid fibrils into less toxic insoluble aggregates (Ono et al., 2004; Wang et al., 2010; Velander et al., 2017). Date palm fruit, a natural source of polyphenols has been found to attenuate oxidative stress, modulate signaling pathways, exert antioxidant properties (i.e., reduce damage due to oxygen), and reduce the risk of AD by enhancing cognitive function in mice (Hartman et al., 2006; Kim et al., 2007; Sathya and Pandima Devi, 2018). For example, dietary supplementation of 2 and 4% date palm fruit (rich in polyphenols) can reduce cognitive deficits and lower Aβ plasma levels in a transgenic mouse model of AD (APP/Tg2576) (Subash et al., 2015). Additional, studies investigating other polyphenol sources, such as curcumin and resveratrol, similarly support the potential neuroprotective effects of polyphenols (Yang et al., 2005; Broderick et al., 2020). When aged AD transgenic mice (Tg2576) are fed curcumin there is an overall reduction in amyloid levels and plaque burden in the brain (Broderick et al., 2020). Likewise, the polyphenolic rich molecule resveratrol decreases neuroinflammation and accumulation of Aβ oligomers, increase levels of synaptic markers, and decrease markers of apoptosis and autophagy in the brains of AD transgenic mice (3xTg-AD) (Yang et al., 2005).

In humans, resveratrol has been assessed in clinical studies and is safe and well-tolerated by patients, and modulates neuroinflammation and influences adaptive immunity (Moussa et al., 2017). However, although polyphenol studies have demonstrated a potential role in preventing and treating dementia it can be difficult to translate results obtained from animal models to humans. For example, in a previous randomized double-blind placebo-controlled trial in AD patients there was no evidence to support resveratrol decreases biomarkers of AD or the ability to clear amyloidosis in the AD brain (Turner et al., 2015). Thus, further studies on human subjects are necessary to reach a better awareness of the role of polyphenols on neurodegenerative diseases.

### Other Dietary Components: Alcohol

Alcohol consumption can influence the development and progression of neurodegenerative diseases and dementia (Thomas and Rockwood, 2001; Ridderinkhof et al., 2002; Oslin and Cary, 2003; Weissenborn and Duka, 2003; Zhu et al., 2004). Research in rodent models of AD have demonstrated that alcohol intake can induce AD-like cognitive deficits in mice and upregulate AD-like pathology (e.g., Aβ 42/40 ratio, total tau) that persist at least as long as 1-month after alcohol consumption has ceased (Hoffman J.L. et al., 2019). In addition, studies show that alcohol use is associated with an increased risk for all types of dementia in humans (Rehm et al., 2019). One study observed that adults in their late 70s, with a history of alcohol abuse (defined as maladaptive pattern of drinking leading to impairment or distress and inability to fulfill expectations), have both cognitive impairment and increased rates of dementia compared to age-matched individuals with no history of alcohol abuse (Thomas and Rockwood, 2001). Broadly speaking, studies demonstrate that alcohol abuse is associated with increased risk of accelerated age-associated cognitive decline and dementia (King, 1986; Ridley et al., 2013). However, it is important to note, there is also evidence to support a protective association between alcohol consumption and age-associated cognitive decline and AD. This positive or negative association between alcohol consumption and cognition and risk of AD is likely due to the type of alcohol consumed (e.g., red wine vs. liquor), pattern of alcohol use (e.g., moderate vs. chronic vs. binge), and age at time of consumption (e.g., adolescence vs. adulthood). For example, research demonstrates that low or moderate red wine consumption may have protective effects on age-related cognitive decline, which may be linked to the polyphenols present in red wine (Peters et al., 2008; Neafsey and Collins, 2011; Granzotto and Zatta, 2014). Additional research is necessary to determine whether alcohol consumption is a risk for developing or preventing AD.

### Dietary Patterns: Western Diet

The Western diet is characterized by consumption of large quantities of saturated/trans-fats, refined carbohydrates, and limited intake of fiber. The Western diet consists of red meat, pre-packaged foods, butter, candy and sweets, fried foods, high-fat dairy products, refined grains, potatoes, corn and high-sugar drinks, and low intake of fruits, vegetables, whole grains, fish, nuts, and seeds. Therefore, the discussions of the various macronutrients already described are also relevant for the discussion of the Western diet.

Consumption of the Western diet is a risk factor for many diseases including AD (Berrino, 2002; Grant, 2016). Western type diets containing large quantities of saturated and trans-fats (i.e., bad fats), refined carbohydrates, and limited intake of fiber are associated with cognitive dysfunction in both animal models and humans (Knopman et al., 2001; Luchsinger et al., 2002; Elias et al., 2003; Molteni et al., 2004; Goldbart et al., 2006; Wu et al., 2006; Parrott and Greenwood, 2007; Farr et al., 2008; Jurdak et al., 2008; Stranahan et al., 2008; Craft, 2009; Yu et al., 2010; Kanoski and Davidson, 2011; Nasreddine et al., 2012; Hebert et al., 2013; Beilharz et al., 2015; Khan et al., 2015). In a retrospective study, consumption of a Western-type diet from childhood through middle age was able to predict cognitive dysfunction in people of advanced age (Hosking et al., 2014). These results demonstrate the detrimental effects of early-life dietary pattern on cognition later in life. Furthermore, a recent population-based, cross-sectional study among 70-year old dementia-free adults found that a higher adherence to a Western dietary pattern is associated with increased pathological total tau levels and a pre-clinical AD biomarker profile (Samuelsson et al., 2021). Thus, long-term consumption of the Western diet appears to promote detrimental age-related cognitive changes in humans.

### Dietary Patterns: Mediterranean Diet

The Mediterranean diet (Medi) has received attention for reducing age-associated cognitive decline and risk of AD (Gardener et al., 2012; Lange et al., 2019; Andreu-Reinón et al., 2021). The Medi diet is comprised of olive oil, assorted fruits, vegetables, cereals, legumes, nuts, moderate consumption of fish, poultry, and red wine; and a low intake of dairy products, red meat, processed meat, and sweets (Fava et al., 2013). The Medi diet is characterized by a beneficial fatty acid profile that is rich in both MUFA and PUFA as well as high levels of polyphenols and antioxidants, high intake of fiber and other low glycemic carbohydrates, and relatively greater vegetable than animal protein intake (Cassani et al., 2017; Makki et al., 2018). Therefore, the discussions of the various macronutrients already described are also relevant for the discussion of the Medi diet.

Retrospective studies have found strong associations between adherence to the Medi diet with decreased incidence of AD (Gardener et al., 2012) and mortality in people who have already been diagnosed with AD (Scarmeas et al., 2007). A recent study obtained brain images from cognitively normal people (30-60 years of age) at baseline and 2-years later after consuming either a Medi or a Western diet (Berti et al., 2018). Brain scans reveal that people who consumed the Medi diet had fewer Aβ deposits and higher evidence of neuronal activity than those consuming the Western diet, which suggests protective effects of the Medi diet (Berti et al., 2018).

Research demonstrating the beneficial effects of increased intake of plant-based foods, and a decrease in the intake of animal products and saturated fats, has spurred more research and dietary trials with plant-based diets in recent years. A hybrid variation of the Medi diet and the DASH (Dietary Approach to Stop Hypertension) diet is called the MIND (Medi-DASH Intervention for Neurodegenerative Delay) diet. This diet emphasizes the consumption of vegetables, berries, nuts, olive oil, whole grains, fish, beans, poultry and a moderate amount of wine. The MIND diet trial recently completed (NCT02817074) and the pending results will shed light on how a Medi style diet impacts AD and age-associated cognitive decline. Previous epidemiological evaluations of existing AD cohorts have shown that adherence to the MIND diet is associated with slower rate of cognitive decline, but the MIND trial is the first prospective study to evaluate the impact of the MIND diet in adults of advanced age (Morris et al., 2015a,b; van den Brink et al., 2019).

The positive effects of Medi-type diets may benefit from combining them with other modalities (e.g., exercise, social interaction). In 2014, a large-scale 2-year study in Finland, called the Finnish Geriatric Intervention Study to Prevent Cognitive Impairment and Disability (FINGER), found benefits of a 2-year combination therapy including physical exercise, a healthy diet, and cognitive stimulation in at-risk elderly people from the general population (Ngandu et al., 2015). Participants in the treatment group were advised to consume a specific diet that included 10–20% of daily energy from proteins, 25–35% daily energy from fat (<10% from saturated plus trans-fatty acids, 10–20% from monounsaturated fatty acids, 5–10% from PUFA (including 2 servings of 5–3 g/day of omega-3 fatty acids), 45–55% daily energy from carbohydrates (<10% from refined sugar), 25–35 g/day of dietary fiber, less than 5 g/day of salt, and less than 5% daily energy from alcohol (Ngandu et al., 2015). Outcomes suggest that a multi-domain intervention can maintain and even improve cognitive function in people of advanced age (Ngandu et al., 2015). These exciting results have prompted additional trials with multi-domain lifestyle-based interventions to examine the impact on cognitive function and risk of cognitive decline among older adults at increased risk of dementia, such as the ongoing United States Study to Protect Brain Health Through Lifestyle Intervention to Reduce Risk (POINTER, NCT03688126) [(National Institutes of Health, 2021) Brain Energy for Amyloid Transformation in Alzheimer’s Disease Study - Full Text View - ClinicalTrials.gov; (National Library of Medicine, 2021). MIND Diet Intervention and Cognitive Decline - Full Text View - ClinicalTrials.gov; (ClinicalTrials, 2021). United States Study to Protect Brain Health Through Lifestyle Intervention to Reduce Risk - No Study Results Posted - ClinicalTrials.gov]. The coming years are likely to provide a plethora of new information.

### Dietary Patterns: Ketogenic Diet

Another popular dietary intervention that may confer neuroprotection is the ketogenic diet. The ketogenic diet is a low-carbohydrate diet characterized by consumption of foods high in fat (particularly long-chain triglycerides) and protein but low in carbohydrates which induces a state of ketosis.

Consumption of a ketogenic diet in a mouse model of AD (APP/PS1) reduces total amyloid levels (Van der Auwera et al., 2005), administration of ketone precursors protects from development of cognitive impairment and reduces levels of amyloid and tau pathologies in a transgenic AD mouse model (3xTg) (Kashiwaya et al., 2013), and administration of ketones (the byproduct of the ketogenic diet) improves cognitive function in wild-type mice (Murray et al., 2016). To date, only a few studies have examined the impact of the ketogenic diet on AD-relevant outcomes in humans but the studies that have been conducted support the neuroprotective effects of the ketogenic diet. A ketone-generating diet improved verbal performance in individuals with mild cognitive impairment and a recent randomized crossover trial demonstrated the ketogenic diet improves daily function and quality of life, two critical factors for people with dementia (Rusek et al., 2019). There are numerous recently completed and ongoing trials that include ketogenic or ketone-generating diets (NCT03130036, NCT0252181818, NCT02984540, and NCT02709356) which will shed additional light on the impact of this dietary pattern on cognitive function and other relevant parameters for AD.

Other modifications of the Ketogenic diet include a diet based on medium-chain triglycerides (MCT). Senile dogs treated with MCT results in reduced amyloid concentrations in the brain (Studzinski et al., 2008). This important finding was supported by a study in humans which demonstrated that oral administration of MCT (which increased ketone levels) were associated with better cognitive function compared to placebo-treated AD-patients or patients with mild cognitive impairment (Reger et al., 2004; Henderson et al., 2009; Ota et al., 2019).

The benefits of a Ketogenic diet or ketogenic type diets may be the consequence of increased consumption of PUFA, like omega-3 fatty acids.

### Summary

The next step is to mechanistically explore how diet may impact the brain. If we can identify converging mechanisms of beneficial or detrimental diets for brain health, this could be exploited to develop new therapies. Although, dietary components (e.g., fat, ketones, polyphenols) can influence the brain directly, there is increasing evidence that indirect effects of diet are also important. The microbiota-gut-brain axis has received an increasing amount of attention in recent years because of accumulating evidence indicating the microbiome influences brain health and neurodegenerative disease (Baumgart et al., 2015; Blazer et al., 2015; Spencer et al., 2017). In this review, we will present literature demonstrating that one critical mechanism by which diet impacts age-associated cognitive decline, dementia, and AD is *via* the intestinal microbiome.

## The Microbiota and Alzheimer’s Disease

The human microbiota are the collection of micro-organisms (e.g., bacteria, fungi, protists, archea, viruses), which inhabit a particular environment. Unique microbiomes are found on the body such as the skin, lungs, and the gastrointestinal tract (GIT). Among these niches the bacteria within the GIT (i.e., the microbiota) have been the most extensively studied. The microbiota consists of commensal (and sometimes pathogenic) bacteria that inhabit mucosal surfaces and the lumen of the GIT. More than 90% of bacterial species in the GIT are composed of Bacteroidetes and Firmicutes phyla, while Actinobacteria, Proteobacteria, and Verrucomicrobia constitute relatively minor proportions (Qin et al., 2010). The microbiota largely have a symbiotic relationship with the host whereby the host provides the microorganisms with a suitable environment and an ample supply of nutrients, while the microbiota perform beneficial activities for the host (AlShawaqfeh et al., 2017; D’Angelo et al., 2018). The microbiota are important for maintenance of optimal health but are also thought to contribute to the promotion of disease (Shreiner et al., 2015; Garcia-Mazcorro et al., 2016; AlShawaqfeh et al., 2017; D’Angelo et al., 2018).

The intestinal microbiota are a highly dynamic community that is impacted by numerous factors like age, diet, and disease (Wilmanski et al., 2021). Indeed, the intestinal microbiota has been implicated in a wide variety of neurodegenerative diseases including multiple sclerosis (MS) (Castillo-Álvarez and Marzo-Sola, 2017), amyotrophic lateral sclerosis (ALS) (McCombe et al., 2020), multiple system atrophy (MSA) (Engen et al., 2017), Parkinson’s disease (PD) (Keshavarzian et al., 2015; Scheperjans et al., 2018), and AD just to name a few (Hill et al., 2014; Jiang et al., 2017; Vogt et al., 2017; Lin L. et al., 2018). While there is no ‘normal’ microbiota, the microbiota composition associated with chronic diseases (such as AD) tends to be characterized by high relative abundance of pro-inflammatory bacteria and pathobionts and low abundance of bacteria that are proposed to be beneficial (so called ‘dysbiosis’). Patients with AD exhibit intestinal microbiota dysbiosis including decreased microbial richness (number of taxa) and diversity (number of different taxa), a low relative abundance of beneficial bacteria with the potential to synthesize short chain fatty acids (SCFA: acetate, propionate, butyrate) as well as higher abundance of taxa that are known to promote or be associated with inflammation (Hill et al., 2014; Jiang et al., 2017; Vogt et al., 2017; Lin L. et al., 2018). Additionally, bacterial nucleic acids are found in post mortem brain tissue of AD patients (indicating that bacteria are able to enter the brain tissue) (Emery et al., 2017). Based on the currently available data, it is not clear if dysbiosis precedes cognitive function, such studies would take decades to conduct. Even if dysbiosis is a consequence of AD (and does not precede the disease), the pro-inflammatory dysbiotic microbiome in AD patients may promote and sustain inflammation that leads to clinical progression of AD. However, there is compelling data demonstrating that intestinal microbiota alterations are sufficient to influence AD-relevant outcomes including brain pathology, structure, function, and behavior. We may be able to exploit this and use strategies to manipulate the microbiota to influence neurodegenerative disease.

### Microbiota Manipulation

The intestinal microbiota can be manipulated with approaches such as fecal microbiota transplants (FMT), administration of probiotics (live beneficial bacteria), intake of prebiotics (products like fiber that promote growth of certain putative beneficial bacterial populations), or antibiotics (to deplete bacteria). The use of these microbiota manipulating approaches can influence the brain.

Manipulation of the intestinal microbiome can influence AD-relevant outcomes in rodent models including behavior, depression, anxiety, memory, AD-like pathology (i.e., Aβ deposition), and the brain’s ability to change and adapt (i.e., neuroplasticity) (Bercik et al., 2011; Gareau et al., 2011; Desbonnet et al., 2014; Catanzaro et al., 2015; Savignac et al., 2015; Luczynski et al., 2016; Chen et al., 2017; Craven et al., 2017; Bonfili et al., 2020; Zhu et al., 2020). For example, studies in germ-free mice show that an absence of a microbiome causes deficits in short-term recognition and working memory, indicating that the microbiota is important for cognitive function (Desbonnet et al., 2014; Luczynski et al., 2016). Indeed, cognitive dysfunction in rats induced by antibiotic treatment (which depletes the microbiome) can be restored by administration of the probiotic *Lactobacillus fermentum* NS9 (Wang et al., 2015). The intestinal microbiome also appears to contribute to the development of the AD phenotype in an AD mouse model. Colonizing an AD mouse model (APP/PS1) with stool from healthy mice (i.e., non-AD model) improves cognitive deficits and reduces brain deposition of Aβ and tau (Sun et al., 2019). Modulating the microbiota with a prebiotic to support the growth of putative beneficial bacteria has a positive impact in multiple rodent models of AD including reduced neuroinflammation, reduced brain pathology, and fewer cognitive deficits. For instance, a recent administration of a novel probiotic formulation, including the lactic acid-producing bacteria *Bifidobacteria* (i.e., SLAB51), to a genetic mouse model of AD (3xTg), was sufficient to mitigate cognitive deficits and reduce Aβ aggregates (Bonfili et al., 2020). Administration of *Bifidobacteria breve* strain A1 reduces neuroinflammation with a concurrent improvement in cognitive function (Kobayashi et al., 2017). Similar beneficial effects on cognitive function are documented after administration of a prebiotic (i.e., fructooligosaccharide) in which consumption of the prebiotic improves memory with a concurrent reduction in oxidative stress and inflammation in the brain, and down-regulation of tau and Aβ expression in an AD transgenic rat model (Chen et al., 2017). These studies support that the intestinal microbiota is sufficient to influence neuroinflammation, AD-like pathology, and cognitive function.

Recent studies and clinical trials have also examined the effect of microbiota manipulation on cognition and other AD-relevant outcomes in humans. Administration of *Bifidobacteria breve* strain A1 improves cognitive function in older adults (Kobayashi et al., 2019). Studies also demonstrate that probiotics (fermented milk products) improve cognition (Camfield et al., 2011; Akbari et al., 2016; Ano et al., 2018, 2019; Athari Nik Azm et al., 2018) and are associated with lower risk of cognitive impairment and dementia (although it should be noted that this finding is not universally recapitulated) (Benton et al., 2007; Ozawa et al., 2013). Studies in AD patients are also promising. A randomized double-blind controlled clinical trial demonstrated that probiotic consumption (*Lactobacillus acidophilus, Lactobacillus casei, Bifidobacterium bifidum, Lactobacillus fermentum*) improves cognitive function in AD patients (Akbari et al., 2016; Tamtaji et al., 2019). Finally, in 2020 a case report was published of an 82-year old AD patient that had undergone 2 months of FMT to treat a *C. difficile* infection. Surprisingly, not only did the FMT effectively treat the *C. difficile* infection, but it also improved cognition (Hazan, 2020). Although, it is possible that elimination of the infection prompted the improvement in cognitive decline, the observation is intriguing. A randomized, double-blind, placebo-controlled trial to evaluate the efficacy of FMT to improve cognitive function in individuals with AD is currently ongoing and will shed light on FMT as a therapeutic approach to treat AD (NCT03822299). These studies support the potential for microbiota-directed treatments for neurodegenerative diseases such as AD.

Additional studies are critically needed to understand the utility of microbiota-directed treatments in AD and age-associated cognitive decline. Diet robustly impacts the intestinal microbiota; therefore it is possible that one mechanism by which diet impacts the brain is by changing the microbiota.

## Diet as a Regulator of the Intestinal Microbiota

Drastic change in diet (e.g., vegetarian to omnivore or vice versa) can rapidly and robustly alter the intestinal microbiota community structure and function within a period of 1-2 days (Goodglass et al., 2001; Wilson et al., 2010; Nasreddine et al., 2012). Diet-induced changes in the intestinal microbiota are the consequence of changes in nutrient availability including amounts and types of macro- and micro-nutrients found in a diet. Below is a discussion of how macronutrients, dietary components, and complex dietary patterns influence the intestinal microbiota composition and profile.

### Dietary Component: Fats

While consumption of fat likely has direct effects on the brain, fat also impacts the intestinal microbiome. Broadly speaking, consumption of “bad fats” such as saturated and trans-fats increases the abundance of pro-inflammatory bacteria while “good fats” such as MUFA and PUFA are associated with an increase of anti-inflammatory SCFA-producing bacteria (Murphy et al., 2015).

Consuming saturated and trans-fats increases the abundance of pro-inflammatory bacteria and reduces the abundance of bacteria that are believed to be beneficial. Studies in rodents show that consumption of a high amounts of saturated and trans-fat in rats reduces the abundance of the putative anti-inflammatory bacteria (*Lactobacillus intestinalis*), and increases the abundance of bacteria generally considered to be pro-inflammatory (*Clostridiales, Bacteroides, Enterobacteriales*) (Lecomte et al., 2015). Similarly, pro-inflammatory bacteria (*Bacteroides, Bilophila*) are increased in mice fed a diet enriched in saturated fat (i.e., lard) (Caesar et al., 2015). Studies in humans show similar pro-inflammatory shifts in the microbiome with consumption of “bad fats.” Specifically, consumption of saturated and trans-fats, increase the abundance of pro-inflammatory bacteria in the phyla Actinobacteria, while concurrently decreasing the abundance of bacteria in the phyla Firmicutes (a phyla that contains numerous bacteria that are thought to be beneficial for health) (Wu et al., 2011; Fava et al., 2013). Moreover, absence of “bad fats” may be sufficient to beneficially modulate the microbiota (even without consuming “good fats”). For example, removing saturated and trans-fat from the human diet increases the relative abundance of putative beneficial bacteria like *Bifidobacterium* (Fava et al., 2013).

In contrast, consumption of PUFA and MUFA, such as extra virgin olive oil, nuts, and flax seeds, have beneficial effects on the intestinal microbiome. One particular study in mice, reports that consumption of fish-derived omega-3 PUFA increases the abundance of probiotic bacteria such as Actinobacteria (*Bifidobacterium* and *Adlercreutzia*), lactic acid-producing bacteria (*Lactobacillus* and *Streptococcus*), and Verrucomicrobia (*Akkermansia muciniphila*) (Caesar et al., 2015). Studies in humans demonstrate the ability of omega-3 PUFA to decrease the Firmicutes:Bacteroidetes ratio (a commonly used index of intestinal health, that represents an imbalance between two dominant phyla) and an increase the abundance of bacterial metabolites (like the SCFA butyrate)-producing bacterial genera, such as *Bifidobacterium, Lachnospiraceae*, and *Roseburia* (Andersen et al., 2011; Balfegó et al., 2016; Watson et al., 2018). Additionally, human studies show that high intake of a MUFA increases the abundance of the putative anti-inflammatory bacterial species such as *Parabacteroides*, and genera *Roseburia* and *Oscillospira*, and correlated to a decrease in pro-inflammatory promoting *Prevotella* (Haro et al., 2016).

Overall, dietary fats have effects on the intestinal microbiome and this could be one way by which diet and fat consumption can influence dementia and AD.

### Dietary Component: Carbohydrates

Simple and complex carbohydrates have different effects on the intestinal microbiome with the former being pro-inflammatory and the later anti-inflammatory.

Long-term intake of a high sugar diet (especially refined sugars) is associated with a pro-inflammatory intestinal microbiota composition including depletion of putative beneficial bacteria resulting in enrichment of pathogenic and pro-inflammatory microbes, as well as a reduction in bacterial diversity in animal studies (Antonini et al., 2019; Satokari, 2020). Specifically, studies in mice demonstrate that high consumption of total daily calories from sugar decreases bacterial diversity, increases the relative abundance of pro-inflammatory Proteobacteria, and simultaneously decreases the abundance of Bacteroidetes (Wolters et al., 2019). Furthermore, fructose and glucose supplementation significantly reduce relative abundance of Bacteroidetes and significantly increase abundance of pro-inflammatory Proteobacteria in mice. In particular, glucose and fructose intake significantly lowered the proportions of *Muribaculum intestinale* (phylum, Bacteroidetes), while there was an increase in *Desulfovibrio vulgaris* (phylum, Proteobacteria) and *Akkermansia muciniphila*, a possible anti-inflammatory bacterium in the intestine (Do et al., 2018). Even consumption of low calorie sweeteners [e.g., sucralose (marketed as Splenda)] (Nettleton et al., 2016; Wang et al., 2018) and natural sugars (e.g., carrageen) can have pro-inflammatory effects on the microbiota in mice, such as a decrease in *Akkermansia muciniphila* (Shang et al., 2017). Although, studies in rodents have thoroughly examined the effects of high-sugar consumption on the intestinal microbiome, studies in humans are more limited due to the level of difficulty to isolate the effect of refined sugars within a complex diet. However, dietary patterns that contain high levels of refined sugar, such as the Western Diet, have a pro-inflammatory effect on the microbiota (discussed below).

Dietary fiber cannot be digested by mammalian enzymes but can be fermented by bacterial enzymes. Consumption of fiber in humans promotes the growth of bacteria that possess the appropriate enzymes and is associated with high microbial richness (generally considered to indicate a “good” microbiota community) (Reddy et al., 1975; Halmos et al., 2015). High fiber intake in humans is associated with higher abundance of putative beneficial bacteria (*Bifidobacterium, Lactobacillus*, and *Ruminococcus, E. rectale, Roseburia*) (Leitch et al., 2007; Costabile et al., 2008; Carvalho-Wells et al., 2010; Walker et al., 2011; Keim and Martin, 2014). Further studies in humans show that high consumption of dietary fibers influences the Firmicute:Bacteroidetes ratio to be more reminiscent of a “healthy” microbiome (De Filippo et al., 2010). Additionally, diets enriched in fermentable plant-based nutrients, such as vegan and vegetarian diets, increase the relative abundance of taxa with potential protective effects including Bacteroidetes, *Prevotella, Roseburia* (Tomova et al., 2019).

In short, high sugar diets are associated with an increased pro-inflammatory intestinal environment whereas diets high in fiber are associated with a microbiota profile that is enriched with putative beneficial bacteria.

### Dietary Component: Proteins

Consumption of high amounts of protein can overwhelm the capacity of the small intestine to digest and subsequently absorb peptides and amino acids, which results in an incomplete digestion and delivery of these proteins to the colon where they are not normally found in high quantities. This encourages the growth of bacteria that are able to use protein as a food source including protein-fermenting bacteria and pathogens that can affect the risk of neurodegenerative diseases (Reddy et al., 1975; De Filippo et al., 2010; Cotillard et al., 2013; David et al., 2014).

In 1977, a culture-based study compared the intestinal microbiota between subjects consuming a high beef diet to subjects consuming a meatless diet, and was the first study to show the effects of dietary protein on the intestinal microbiota (Hentges et al., 1977). This study demonstrated that consuming a diet high in beef was sufficient to lower the relative abundance of commensal anti-inflammatory bacteria (*Bifidobacterium adolescentis*) and increase the relative abundance of pro-inflammatory bacteria (*Bacteroides* and *Clostridia* species) in the intestinal microbiome (Hentges et al., 1977). Further studies in humans also observe that consumption of animal-based proteins increases the abundance of bile-tolerant anaerobes (*Bacteroides, Alistipes, Bilophila*), which can have pro-inflammatory effects (Reddy et al., 1975; Cotillard et al., 2013; David et al., 2014). Thus, diets enriched in animal protein leads to an unfavorable decrease in the beneficial bacteria and increase in potentially disease-inducing, pro-inflammatory bacteria.

It is important to note that it is challenging to isolate the impact of animal protein on the microbiota since fat content is also a feature of red meat. However, not all proteins are the same. Consumption of plant-based protein like whey and pea extract in humans increase the endogenous probiotic bacteria *Bifidobacterium* and *Lactobacillus* and decrease dangerous and pathogenic bacteria like *Bacteroides fragilis* and *Clostridium perfringens* (Reddy et al., 1975; Romond et al., 1998; Dominika et al., 2011). Overall, further studies that examine the isolated effects of plant-based protein in both animal models and humans are needed to fully comprehend the benefits of plant-based protein on the intestine. However, dietary patterns that contain high plant-based nutrients, such as the Mediterranean diet are beneficial (discussed below).

Protein clearly impacts the intestinal microbiome. However, whether protein intake encourages an environment of pathogens and protein-fermenting bacteria that increases the risk of disease or a beneficial and anti-inflammatory intestinal milieu, is dependent on the type of protein consumed.

### Other Dietary Components

There are an incredibly large number of individual dietary components that could be discussed; however, we have limited our discussion to those that have been linked to specific dietary patterns or with compelling epidemiological evidence. Namely, we will discuss polyphenols (an important part of the Medi diet) and alcohol.

### Other Dietary Components: Polyphenols

As previously discussed, polyphenols are micronutrients with anti-oxidant properties that naturally occur in plant and plant-based foods like fruits, seeds, and vegetables (Pérez-Jiménez et al., 2010). Extensive analysis of a variety of human studies suggest that polyphenols can affect the intestinal microbiota community, affecting microbial richness, diversity, and composition (Singh et al., 2019). For example, studies show that curcumin, a polyphenol-rich substance, can significantly increase the abundance of butyrate-producing bacteria and reduce the load of pathogenic bacteria such as *Prevotellacceae, Coriobacterales, Enterobacteria*, and *Enterococci* in rodents (Zam, 2018). Additionally, studies in humans demonstrate that consumption of polyphenol-containing foods (e.g., date palm fruit, flavanoids, wine, green tea, cocoa, grapeseed extract, and pomegranate) promote a beneficial intestinal microbiome profile that is composed of *Bifidobacterium* and *Lactobacillus*, and lower levels of pathobionts including *Staphylococcus aureus, Salmonella typhimurium*, and *Clostridium* species (*C. perfringens* and *C. histolyticum*) (Lee et al., 2006; Parkar et al., 2008; Tzounis et al., 2008, 2011; Bialonska et al., 2010; Vendrame et al., 2011; Jin et al., 2012; Queipo-Ortuño et al., 2012; Cueva et al., 2013; Cuervo et al., 2014; Druart et al., 2014; Eid et al., 2014).

### Other Dietary Components: Alcohol

Alcohol consumption can be either beneficial or harmful for brain health including age-associated cognitive decline and AD which could be due to types of alcohol consumed and pattern of alcohol use. Additionally, red wine contains polyphenols, which have already been discussed, and generally induce beneficial changes to the microbiota. Indeed, consumption of red wine is reported to increase the relative abundance of putative good bacteria (e.g., *Bifidobacterium*) (Queipo-Ortuño et al., 2012). However, problematic drinking (binge, alcohol use disorders) is consistently reported to be detrimental for brain health and not surprisingly these patterns of alcohol consumption are associated with detrimental changes in the intestinal microbiome (dysbiosis). Alcohol consumption in rodents induces a decrease in the abundance of bacteria thought to be beneficial (phyla Firmicutes) and an increase in putative pro-inflammatory bacteria (Bacteroidetes, Proteobacteria, Verrucomicrobia) (Keshavarzian et al., 2009; Mutlu et al., 2009, 2012). Furthermore, alcohol administration to rats induces intestinal bacterial overgrowth (an abnormal increase in the microbiota population) characterized by an enrichment of pro-inflammatory Gram-negative bacteria (*E. Coli, Enterococci, Klebsiella, Pasteurella, Proteus*, and *Pseudomonas, Shigella*) (Yan and Schnabl, 2012).

These studies have been largely recapitulated in humans. Recently, a human study demonstrated that compared to individuals with low (or no) history of alcohol intake, chronic alcohol consumption is associated with a more pro-inflammatory microbiota profile including high abundance of pro-inflammatory bacteria [e.g., *Clostridium, Holdemania* (Firmicutes), and *Sutterella* (Proteobacteria)] and with a decreased abundance of putative beneficial bacteria (e.g., *Faecalibacterium* genus) compared to non-chronic alcohol consumers (Bjørkhaug et al., 2019). Taken together, alcohol consumption (especially problematic drinking) increases pro-inflammatory bacteria with a concurrent decrease in bacteria thought to be beneficial; however, type of alcohol consumption (such as red wine) might override some of the negative effects of alcohol consumption.

### Special Diets: Western, Mediterranean, and Ketogenic

The Western diet is well established to cause pro-inflammatory changes in the intestinal microbiome (García-Montero et al., 2021). Specifically, in humans, consumption of a Western diet is associated with high abundance of pro-inflammatory microbiota (e.g., Proteobacteria, *Bacteroides*), an increase in the Firmicute:Bacteroidete ratio (generally considered to be pro-inflammatory), and bacterial overgrowth (Wu et al., 2011; Shankar et al., 2017). Additionally, the Western diet also is associated with reduced relative abundance of putative beneficial bacteria that include *Bfidobacterium* and *Eubacterium* in children (Shankar et al., 2017). In combination, these changes result in a pro-inflammatory microenvironment and indeed consumption of a Western diet is associated with high levels of systemic inflammation compared to healthy dietary patterns (Noble et al., 2017; Christ et al., 2018).

Conversely, adherence to the Medi diet is associated with a healthy intestinal microbiota. In humans, the intestinal microbiota profile associated with the Medi diet consists of a high abundance of SCFA-producing bacteria [Firmicutes such as *Roseburia, Lactobacillus*, and *Bifidobacterium* (Actinobacteria)], and low abundance of pro-inflammatory bacteria [*Prevotella* (Bacteroidetes), *Clostridium* (Firmicutes)] (Bialonska et al., 2010; Queipo-Ortuño et al., 2012; Berti et al., 2018; Garcia-Mantrana et al., 2018). Additionally, the Medi diet is characterized by high fiber content and (not surprisingly) consumption of the Medi diet is associated with high abundance of the beneficial fiber-fermenting bacteria (*Faecalibacterium prausnitzii*), which is followed by a reduction in systemic inflammation in humans (Meslier et al., 2020). Furthermore, a recent clinical study found that consuming the Medi diet for 1 year is sufficient to beneficially alter the intestinal microbiome and promote the growth of bacteria that are negatively associated with inflammation and frailty as well as improved cognitive function (Ghosh et al., 2020). Thus, consumption of the Medi diet has beneficial effects on the microbiota which can have an important biological impact.

Consumption of the Ketogenic Diet is also associated with changes in the microbiota. The low level of carbohydrates reduces the abundance of bacteria that preferentially use carbohydrates as a food source (Paoli et al., 2019). Studies in humans, find that consumption of a Ketogenic diet reduces the abundance of putative beneficial bacteria (e.g., Firmicutes, *Bifidobacteria, E. rectale, Dialister, Roseburia, Bacteroides, Faecalibacterium prausnitzi*) whereas pro-inflammatory bacteria are increased (e.g., *E. Coli, Desulfovibrio* spp., Bacteroidetes) (Swidsinski et al., 2017; Tagliabue et al., 2017; Zhang et al., 2018; Lindefeldt et al., 2019). Similarly, another study found that subjects who consume a high protein, low carbohydrate diet have reduced abundance of putative beneficial bacteria (*Roseburia, Eubacterium rectale*) (Russell et al., 2011). These changes are not typically considered to be a “beneficial” profile and yet the Ketogenic diet has a positive impact on cognition as already discussed. However, a study has demonstrated that the Ketogenic diet reduces the abundance of Proteobacteria, which may be important in AD (Xie et al., 2017). Clearly, additional studies are needed to fully understand the impact of the Ketogenic diet on the microbiome.

### Summary

Dietary patterns and components influence the intestinal microbiota which are summarized in Supplementary Table 1. These diet-induced changes in intestinal microbiota signatures may influence cognitive function and risk of AD through a variety of mechanisms which are discussed below.

## Mechanisms of Communication – the Microbiota-Gut-Brain Axis

There are many mechanisms by which the intestinal microbiota can influence the brain and AD (Carabotti et al., 2015; Ma et al., 2019). The list below is by no means all-encompassing (and it is highly likely that several mechanisms are contributing simultaneously) but this list represents some of the most compelling and plausible mechanisms that have been implicated to date.

### Bacterial Components and the Immune System

Bacteria have surface components that can elicit robust immune responses. One of the most well studied of the pro-inflammatory bacterial components is lipopolysaccharide (LPS) which is found in the outer membrane of Gram-negative bacteria. The abundance of bacteria containing LPS (e.g., *Alistipes, E. coli, Bacteroides fragilis*) are increased in AD patients and have the potential to elicit a pro-inflammatory immune response and lead to neuroinflammation (Vogt et al., 2017; Parker et al., 2020). Another well-studied bacterial toxin that influences neuroinflammation is lipoteichoic acid (LTA), a cell wall component in Gram-positive bacteria with similar pro-inflammatory qualities as LPS (Huang et al., 2013; Howe et al., 2020).

Typically, the pro-inflammatory contents of the intestine (including bacteria containing LPS and LTA) are maintained within the lumen of the GIT by the intestinal barrier which is formed of mucus, anti-microbial peptides, and a layer of epithelial cells linked together by the apical junctional complex and tight junction proteins (Martin et al., 2018). However, disruption of the barrier (called intestinal hyper-permeability or leaky gut) permits entry of pro-inflammatory bacteria and molecules like LPS and LTA into the intestinal mucosa, systemic circulation, and organs throughout the body (including the brain) where they can elicit profound immune activation. The integrity of the intestinal barrier is influenced by numerous factors including the intestinal microbiota (Martin et al., 2018). Thus, microbiota dysbiosis (triggered by diet or disease) can induce intestinal barrier dysfunction allowing bacteria and bacterial components to reach the intestinal mucosa and systemic circulation where they can trigger a pro-inflammatory immune response.

For years it was believed that the brain was an immune privileged organ, meaning that immune function in the periphery had no direct impact on the brain; however, we now know this is not the case. Immune cells in the periphery are influenced by the intestinal microbiota, can cross the blood brain barrier (BBB), and are found in post mortem brain tissue (Papotto et al., 2021). Brain tissue from a rodent model of AD and AD patients demonstrate that innate (e.g., macrophages, monocytes, neutrophils) and adaptive (i.e., T cells) immune cells can infiltrate the CNS and accumulate near areas of Aβ and tau pathology (Fiala et al., 2002; Baik et al., 2014; Merlini et al., 2018; Gate et al., 2020). The peripheral immune system is impacted by the intestinal microbiota so this finding could be a critical mechanism by which the microbiota influences the brain and AD (Zheng et al., 2020).

Accumulation of Aβ oligomers may be a response to LPS exposure. Oligomers of Aβ have potent, broad spectrum antimicrobial properties by forming fibrils that entrap pathogens and disrupt cell membranes. Thus, Aβ accumulation could be a consequence of long-term dysbiosis and exposure to bacterial components such as LPS (Spitzer et al., 2010, 2016; Wang et al., 2014; Kumar et al., 2016; Zhan et al., 2018). Indeed, there is increased abundance of Gram-negative bacteria that contain LPS in the AD brain which is co-localized with Aβ (Zhan et al., 2016, 2018; Zhao et al., 2017a,b).

Finally, resident innate immune cells within the CNS, such as microglia, have been widely studied in AD and are thought to be critically important. Microglia provide many critical functions for brain homeostasis including removal of pathogens, cell debris, and misfolded proteins which is typically characterized by release of inflammatory mediators, such as cytokines and chemokines, and generation of reactive oxygen and nitrogen species (Lucas et al., 2006; Liddelow et al., 2017; Milatovic et al., 2017). In fact, *in vitro* studies demonstrate microglia have the potential to remove accumulated Aβ and tau plaques (Paresce et al., 1996; Koenigsknecht, 2004; Lai and McLaurin, 2012; Bolós et al., 2015). However, uncontrolled and/or prolonged activation of microglia can lead to chronic neuroinflammation, which thought to contribute to the development and progression of neurodegeneration and AD (Tahara et al.; Lucas et al., 2006; Gambuzza et al., 2014; Liddelow et al., 2017). Bacteria in the brain (or other microbiota-mediated mechanisms discussed below) due to intestinal barrier dysfunction or activation of microglia by Aβ or tau could be sufficient to trigger chronic microglial activation. Ultimately, collective findings suggest chronic microglial activation could be the consequence of intestinal microbiota dysbiosis (Abdel-Haq et al., 2019).

Taken together there are multiple mechanisms by which the microbiome can promote neuroinflammation and brain pathology leading to cognitive dysfunction and AD.

### Bacterial Metabolites

The intestinal microbiota can influence the brain, directly or indirectly, through the production of a wide variety of microbial-derived metabolites (Den Besten et al., 2013; Dando et al., 2014; Keshavarzian et al., 2015; Zhan et al., 2018; Ghosh et al., 2020; Tulkens et al., 2020). Below are a few examples of the bacterial metabolites that appear to be important for age-associated cognitive decline and AD.

Synthesize short chain fatty acids (e.g., acetate, butyrate, propionate) are a metabolic byproduct of fiber fermentation by certain types of bacteria (e.g., Firmicutes: *Roseburia, Bifidobacterium, Lactobacillus, Ruminococcus*, and *E. rectale*) (Duncan et al., 2004; Tan et al., 2014). SCFA can cross the BBB to influence the brain directly since neurons and microglia have receptors and transporters for SCFA (Oldendorf, 1973). Additionally, SCFA have numerous biological effects that can influence the brain indirectly including effects on integrity of the intestinal and BBB as well as the immune system and neuroinflammation (Salminen et al., 1998). SCFA (especially butyrate) are used as an energy source for epithelial cells that make up the intestinal barrier (low butyrate is associated with intestinal barrier dysfunction) and regulate the immune system (generally, although not exclusively, thought to be anti-inflammatory) (Schirmer et al., 2016). The effects of SCFA are thought to be mediated by G-protein coupled receptors (GPCR). For example, SCFA can bind to GPCR located on immune cells, and numerous other cell types in the brain, to directly impact cellular signaling (Dalile et al., 2019). SCFA (especially butyrate) can also be transported into cells (*via* monocarboxylate transporters) where they act as histone deacetylase (HDAC) inhibitors and impact gene expression and consequently cell function (Kien et al., 2008; Bailón et al., 2010; Liu et al., 2012; Vinolo et al., 2012; Chang et al., 2014). Inhibition of HDAC activity, promotes protective epigenetic modulations in multiple cell types including the intestine, immune cells, and the CNS (Stilling et al., 2016). AD patients have increased expression of a particular HDAC (i.e., *HDAC6*) in the hippocampus and other relevant brain regions compared to age matched controls; (Bassett and Barnett, 2014) therefore, approaches that blunt HDAC activity (e.g., with bacterial-derived SCFA) may be clinically impactful. Taken together, there are numerous ways in which SCFA may be important in mediating microbiome-gut-brain axis signaling (Cryan et al., 2019).

Trimethylamine N-oxide (TMAO) is another bacterial metabolite of interest for AD. TMAO is a proatherogenic compound that increases risk of cardiovascular disease and may also contribute to neurodegenerative disease (Wang et al., 2011; De Filippis et al., 2016; Arrona Cardoza et al., 2021). Consumption of carnitine and choline rich foods (typically red meat and dairy) are associated with high levels of TMAO (Koeth et al., 2013). TMAO is produced by the conversion of carnitine and choline to TMA by intestinal bacteria expressing certain enzymes (i.e., CutC/D) which is then converted to TMAO by the enzyme flavin-containing monooxygenase 3 (FMO3) (Akerman et al., 1999; Cashman and Zhang, 2006; Craciun and Balskus, 2012; Bennett et al., 2013; Brown and Hazen, 2015). Alzheimer’s disease patients have elevated levels of TMAO (del Rio et al., 2017). The elevated levels of TMAO might be particularly detrimental as a recent study in mice demonstrates that TMAO can cause deleterious changes in the brain (e.g., neuronal senescence, mitochondrial damage) and promote accelerated brain aging (Li et al., 2018). Accordingly, a computational analysis of publicly available human databases [e.g., The Human Metabolome Database (HMDM), Search Tool for Interactions of Chemicals (STITCH), Genome-Wide Association Studies (GWAS), Search Tool for the Retrieval of Interacting Genes/Proteins (STRING), Molecular Signatures Database (MSigDB)] has identified multiple common genetic pathways between TMAO metabolism and AD biomarkers (Xu and Wang, 2016). These genes include, but are not limited to, pathways involved in the adaptive immune system, axon guidance, and metabolism of proteins, lipids and lipoproteins. Moreover, TMAO may influence Aβ and tau (Scaramozzino et al., 2006). Thus, the association between TMAO and AD, highlights the importance of microbial metabolites the brain and potentially neurodegenerative disease.

Lastly, several microbial metabolites produced through tryptophan metabolism can influence the brain. The kynurenine pathway is a route of tryptophan catabolism that produces a number of biologically active metabolites such as nicotinamide adenine dinucleotide (NAD^+^), kynureinine, kynurenic acid, quinolinic acid, and 3-hydroxykynurenine, 5-hydroxyindole-acetic acid (5-HIAA), and picolinic acids (Zwilling et al., 2011; Maitre et al., 2020). Studies in rodents demonstrate that tryptophan-enriched diets have protective effects against hippocampal β-amyloid accumulation in mouse model of AD (Noristani et al., 2012) and improved memory (Musumeci et al., 2017). Metabolites in the kynurenine pathway have been linked to beneficial effects on the brain: 5-HIAA decreases Aβ oligomer accumulation and improves memory and kynurenic acid counteracts Aβ-induced neurotoxicity, and prevent neuronal/synaptic loss and memory deficits in animals (Zwilling et al., 2011; Maitre et al., 2020). However, the data in humans suggests that the kynurenine pathway may be detrimental. Quinolinic acid is up-regulated in AD patient brain tissue which is important since quinolinic acid causes lipid peroxidation and is neurotoxic (*via* activity as a N-methyl D-aspartate (NMDA) receptor agonist) (Guillemin et al., 2005). Additionally, another study reports that the kynurenine/tryptophan ratio is associated with inflammation and impaired memory in humans (Willette et al., 2021). Clearly, the impact of metabolites in the kynurenine pathway on AD is complex (and likely depends on the specific metabolite) highlighting the need for additional studies.

### Neurotransmitters

Neurotransmitters are endogenous chemicals that allow neurons to communicate with each other. Several bacterial strains can independently synthesize (or modulate the synthesis of) a number of neurotransmitters, including γ-aminobutyric acid (GABA), 5-hydroxytryptamine (5-HT, serotonin), dopamine, and noradrenaline (Khan et al., 2015; Fung et al., 2017; Calvani et al., 2018; Sherwin et al., 2018; Strandwitz, 2018). Alteration in the levels of neurotransmitters are observed in various neurological disorders, including Parkinson’s disease and AD (Francis, 2005; Nutt, 2008; Barone, 2010; Brisch et al., 2014). While these changes likely reflect neuronal loss, it is possible that peripheral production of neurotransmitters by bacteria could contribute. Intestinal enterochromaffin cells produce approximately 95% of 5-HT, and can be influenced by microbiota and microbiota metabolites (Liu et al., 2021). Morphologically, enterochromiffin cells are distinct between germ-free and specific pathogen-free mice (Uribe et al., 1994). In fact, levels of serum 5-HT are reduced in germ-free mice and antibiotic-treated mice (Wikoff et al., 2009; Sjögren et al., 2012; Yano et al., 2015). The bacterial influence on 5-HT production may not be trivial as 5-HT can influence the transcription of proteins that modulate Aβ clearance, which have been associated to AD pathogenesis (Maitre et al., 2020). While there is evidence potentially linking bacterial production of neurotransmitters to AD, it is still a matter of debate.

### Bacterial Modification of Host-Produced Compounds

The host and the intestinal microbiota co-produce a large array of small molecules, many of which play a critical role in the health. During food metabolism, the host produces a multitude of compounds that are subsequently modified by the microbiota. The liver produces primary bile acids which aid in the absorption of dietary fat (Monteiro-Cardoso et al., 2021). Most bile acids are absorbed in the small intestine, concurrently with the absorption of lipids, however a small amount of unabsorbed primary bile acids can reach the colon where the intestinal microbiota convert them to secondary bile acids which are subsequently absorbed (Monteiro-Cardoso et al., 2021). These secondary bile acids can cross the BBB and are neuroactive (Monteiro-Cardoso et al., 2021). Recent evidence suggests a link between cognitive impairment and increased levels of the bacterially produced, secondary bile acids (i.e., deoxycholic acid, glycodeoxycholic, taurodeoxycholic, and glycolithocholic) in AD patients (MahmoudianDehkordi et al., 2019). There is no doubt that much will be revealed in the coming years about how secondary bile acids contribute to cognition, dementia, and AD.

### Extracellular Vesicles

Extracellular vesicles (EV) are membrane vesicles that are released from numerous cell types, including intestinal epithelial cells (exosomes) and bacteria (outer membrane vesicles (OMV), membrane vesicles) (András and Toborek, 2016; Van Niel et al., 2018; Doyle and Wang, 2019). EV contain toxins, nucleic acids, proteins (including cytokines), and genetic material and are capable of crossing the BBB and influencing neuroinflammation and brain function (Cuesta et al., 2021). Compared to age-matched controls, cognitively impaired and AD patients have a different EV profile including increased levels of proinflammatory cytokines (i.e., pro-IL-1β, IL-6, IL-10, TNF-α) (Chan et al., 2021). Additionally, AD patients have a significantly different metabolite profile within in bacterial OMV (e.g., aspartate, L-aspartate, L-glutamate) (Wei et al., 2019). These differences appear to be biologically relevant. OMV derived from AD patients can cross the BBB, promote microglia activation and inflammation, promote tau hyper-phosphorylation, and impair cognitive function when administered to wild-type mice (Wei et al., 2020). The study of EV in AD are still in their infancy, and there is clearly much to be learned about the influence of EV on cognitive function, dementia, and AD.

### Neuroendocrine Signaling

There are numerous cell types within the intestinal mucosa that are responsive to changes in bacterial-derived metabolites. Perhaps chief among them are enteroendocrine cells which produce glucagon-like peptide-1 (GLP-1) and cholecystokinin (CCK) (Heitz, 1979; Latorre et al., 2016; Farzi et al., 2018).

Synthesize short chain fatty acids and secondary bile acids, promote the production of GLP-1 (Tolhurst et al., 2009, 2012; Bodnaruc et al., 2016; Koh et al., 2016). GLP-1 can cross the BBB can impact neurons and microglia *via* GLP-1 receptors on these cell types (Hölscher, 2014; Katsurada and Yada, 2016; Kim et al., 2017). In both AD animal models and patients with AD, GLP-1 improves neural plasticity and protects against neuronal apoptosis and neuroinflammation (Li et al., 2012; Hölscher, 2014; Anderberg et al., 2016). Indeed, mice with a deficit in GLP-1 receptors demonstrate impaired performance in memory-related behavioral tasks (Hölscher, 2014). Thus, levels of microbiome-derived SCFA and secondary bile acids can influence brain function *via* neuroendocrine signaling such as GLP-1.

Cholecystokinin is another neuropeptide produced by enteroendocrine cells that can cross the BBB (Blevins et al., 2000). The CCK receptor (i.e., CCK-B receptor), is highly expressed in brain regions such as the hippocampus (Dockray et al., 1978; Innis et al., 1979; Zarbin et al., 1983; Braak et al., 1993; Pietrowsky et al., 1994) and studies demonstrate that administration of CCK-B receptor agonists injected directly into the hippocampus improve memory (Sebret et al., 1999). Accordingly, low expression levels of CCK are associated with impaired memory in aged rodents (Croll et al., 1999). Thus, CCK possess a critical role in cognitive impairment and AD (Plagman et al., 2019).

Levels of GLP-1 and CCK are influenced by the intestinal microbiota and is one mechanism by which the microbiota may influence the brain.

### Vagus Nerve

In addition to the systemically mediated mechanisms discussed above, the GIT is physically connected to the brain through the vagus nerve. The vagus nerve is part of the parasympathetic nervous system and has a composition of 80% afferent and 20% efferent fibers (Agostoni et al., 1957; Bonaz et al., 2018). Vagal signaling influences inflammation and the production of brain derived neutrotrophic factor (BDNF) (Bonaz et al., 2018; Breit et al., 2018). Bacterial components like LPS, bacterial metabolites like SCFA, and products produced by enteroendocrine cells (e.g., 5-HT) or enteroendocrine cells (i.e., CCK) (both influenced by the microbiota) influence the vagus nerve (Bonaz et al., 2018). For example, *in vivo* and *ex vivo* studies demonstrate that *Lactobacillus johnsonii* influences vagal nerve activity (Tanida et al., 2005). The regulation of vagal nerve activity by the microbiota may be critically important. Vagus nerve stimulation (through non-invasive electrical current) in animal models of AD results in morphological (e.g., number of branches, branch length) changes in microglia—specifically, it induces a change from a neuro-destructive to a neuroprotective phenotype (Kaczmarczyk et al., 2018). Vagotomy alters microglia (and astrocyte) morphology (Hofmann et al., 2021). Additionally, vagus nerve ablation in mice is sufficient to prevent *Lactobacillus rhamnosus*-induced changes in rodent behavior (Bravo et al., 2011). Despite these compelling data in rodents, a retrospective human did not find a significant association between vagus nerve ablation and cognitive function (Lin S.Y. et al., 2018). Thus, additional studies are warranted in order to better understand the direct role of the vagus nerve in brain health, cognition, and AD.

### Other Considerations: Time of Food Consumption

Circadian rhythms are biological rhythms (regulated by a molecular circadian clock that is present in mammalian and bacterial cells) that are approximately 24 h that optimize biological functions with the external environment (Voigt et al., 2016, 2019). There is an increasing evidence demonstrating that intestinal bacteria community structure and function fluctuate across 24 h (Thaiss et al., 2014, 2016; Leone et al., 2015; Liang et al., 2015). In fact, there are striking diurnal rhythms in the expression of bacterial genes including genes important for metabolism. Thus, consumption of the same food products at different times of the day could result in production of different metabolic byproducts (e.g., SCFA), EV, or even the capacity of enteroendocrine cells to produce GLP-1. Consequently, when you eat could influence the mechanisms by which diet *via* the microbiome affects the brain.

### Summary

There are many ways that the intestinal microbiome can influence the brain and neurodegenerative diseases. Likely, several of these mechanisms are working in tandem to concurrently influence the microbiome effects on neuroinflammation, cognition, dementia, and AD.

## Conclusion

**FIGURE 3:**
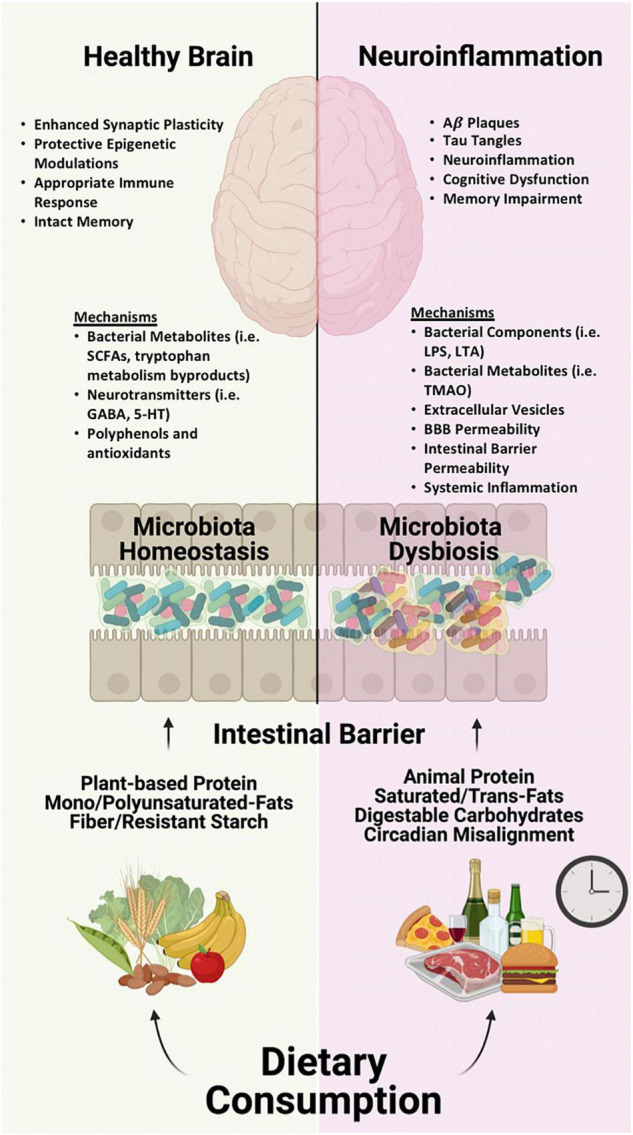
The composition and patterns of dietary intake influence the intestinal microbiome environment, which regulate different mechanisms of communication throughout the microbiota-gut-brain axis, ultimately impacting brain health. **Healthy Brain:** A diet rich in plant-based protein, mono/polyunsaturated fats, and fiber/resistant starches induce an anti-inflammatory microbiome, which elicits protective mechanisms of communication that protect the brain against neurodegeneration. **Neuroinflammation:** A diet consisting of high quantities of animal protein, saturated/trans-fats and carbohydrates, along with circadian misalignment influence a pro-inflammatory microbiome and induce adverse mechanisms of communication which can lead to neuroinflammation and neurodegenerative disease such as AD.

Although, many factors contribute to the onset of AD (e.g., genetics, lifestyle), evidence strongly supports a potential impact of diet on the development/progression of cognitive function, brain health, and neurodegeneration. Studies, in both animal models and humans, demonstrate that diet influences AD pathology, behavior/cognitive function, and risk of age-associated cognitive decline and AD. Diet robustly and rapidly influences the intestinal microbiome, and diet-induced changes in the intestinal microbiome could be one important mechanism by which diet influences the brain. Critical mechanisms of communication of the microbiota-gut-brain axis include: (1) the exposure of the brain to microbiota components (e.g., LPS, LTA), metabolites (e.g., SCFA, TMAO, neurotransmitters), and other products (e.g., EV) that can elicit an immune response and/or cross the BBB; (2) Microbiota-modification and metabolism of host products (e.g., bile acids); (3) secondary pathways affected by microbiota metabolism and byproducts (e.g., neuroendocrine signaling, vagus nerve) (Figure 3). These mechanisms are an active area of research by many groups and the upcoming years are going to be highly informative about the relative contributions of these mechanisms on cognitive function, dementia, and AD.

Evidence indicates that humans with AD have microbiota dysbiosis. Whether changes in the microbiota precede the onset of brain pathology and/or behavioral abnormalities is a question that continues to plague the field. Determining the time course of microbiota changes versus neurodegeneration in humans will take decades to resolve, however, such an investment would be worth the effort. Nonetheless, there is an overwhelming amount of data to suggest that pro-inflammatory changes in the microbiome can play a critical role in promoting neuroinflammation and the clinical progression of AD.

Large scale studies combining the efforts of multiple clinical trials are necessary to identify if there are overlapping features that predict response to diet and/or development of cognitive decline. It is only with additional such studies that we will be able to understand the complex relationship between the intestinal microbiota and the brain.

Microbiota-directed treatments (like diet, prebiotics, or probiotics) may be a promising therapeutic approach to influence development and progression of AD. Although consuming a healthy diet is beneficial for the brain, to date no dietary intervention that has been explicitly shown to prevent/treat AD or cognitive decline. This outcome may be due to additional factors that determine individual response to diet. This variability is not yet well understood but differences in the intestinal microbiome could be important. Going forward, personalized medicine may be an important consideration for recommendations (e.g., MIND vs. ketogenic diet) to prevent or delay cognitive decline and/or neurodegeneration.

## Author Contributions

All authors wrote the first draft and contributed significantly to the writing of the review, edited and approved the final draft, and finally revised resubmission.

## Conflict of Interest

The authors declare that the research was conducted in the absence of any commercial or financial relationships that could be construed as a potential conflict of interest.

## Publisher’s Note

All claims expressed in this article are solely those of the authors and do not necessarily represent those of their affiliated organizations, or those of the publisher, the editors and the reviewers. Any product that may be evaluated in this article, or claim that may be made by its manufacturer, is not guaranteed or endorsed by the publisher.
