# Supplementary material for: Dietary Regulation of Gut-Brain Axis in Alzheimer’s Disease: Importance of Microbiota Metabolites
Source: Front Neurosci. 2021 Nov 19;15:736814. doi: 10.3389/fnins.2021.736814 (PMC8639879; doi:10.3389/fnins.2021.736814)
Supplement: Supplementary file 1 [file Data_Sheet_1.PDF]

Table 1. Dietary Consumption Pattern in Relationship to Intestinal Bacterial Composition:

Diet Pattern / Dietary Component	Impact on Cognitive Function	Bacterial Composition (↑ increased; ↓ decreased)	References
**Western Diet**	Associated with cognitive impairment	• **Actinobacteria phylum:**<br>↓ *Bfidobacterium*<br>• **Bacteroidetes phylum:**<br>↑ *Bacteroides*<br>• **Firmicute phylum:**<br>↓ *Eubacterium*<br>▪ ↑ **Proteobacteria phylum:**<br>• ↑ **Firmicutes:Bacteroidetes** ratio	• Drasar, B. S. *et al.* (1973)<br>• Wu, G. D. *et al.* (2011)<br>• Shankar, V. *et al.* (2017)<br>• García-Montero, C. *et al.* (2021)
**Mediterranean Diet**	Associated with improved cognitive function	▪ **Actinobacteria phylum:**<br>↑ *Bifidobacterium*<br>▪ **Bacteroidetes phylum:**<br>↓ *Prevotella*<br>▪ **Firmicutes phylum:**<br>↑ *Roseburia*<br>↑ *Lactobacillus*<br>↑ *Faecalibacterium prausnitzii*<br>↓ *Clostridium*	• Bialonska, D. *et al.* (2010)<br>• Furet, J. P. *et al.* (2010)<br>• Queipo-Ortuño, M. I. *et al.* (2012)<br>• Bifulco, M. (2015)<br>• Meslier, V. *et al.* (2020)<br>• García-Montero, C. *et al.* (2021)<br>• Wang, D. D. *et al.* (2021)
**Ketogenic Diet**	Associated with some improvement of cognitive function	▪ ↓ **Firmicutes**<br>↓ *Eubacterium rectale*<br>↓ *Dialister*<br>↓ *Roseburia*<br>↓ *Faecalibacterium prausnitzi*<br>↓ *Eubacterium rectale*<br>▪ **Actinobacteria phylum:**<br>↓ *Bifidobacteria*<br>▪ ↑ **Bacteriodetes phylum:**<br>↓ *Bacteroides*<br>▪ **Proteobacteria phylum:**<br>↑ *Escherichia Coli*<br>↑ *Desulfovibrio*	• Paoli, A. *et al.* (2019).<br>• Lindefeldt, M. *et al.* (2019).<br>• Swidsinski, A. *et al.* (2017).<br>• Tagliabue, A. *et al.* (2017).<br>• Zhang, Y. *et al.* (2018).<br>• Russell, W. R. *et al.* (2011).
**Fat:<br>Saturated- and Trans-fat**	Associated with cognitive impairment	▪ ↑ **Actinobacteria phylum**<br>▪ **Bacteroidetes phylum:**<br>↑ *Bacteroides*<br>▪ ↓ **Firmicutes phylum:**<br>↓ *Lactobacillus intestinalis*<br>↑ *Clostridiales*<br>▪ **Proteobacteria phylum:**<br>↑ *Enterobacteriales*<br>↑ *Bilophila*	• Wu, G. D. *et al.* (2011)<br>• Fava, F. *et al.* (2013)<br>• Lecomte, V. *et al.* (2015)<br>• Caesar, R., *et al.* (2015)
**Fat:<br>Mono- and Poly-unsaturated**	Associated with improved cognitive function	▪ **Actinobacteria phylum:**<br>↑ *Bifidobacterium*<br>↑ *Adlercreutzia*<br>▪ **Bacteroidetes phylum:**<br>↑ *Parabacteroides*<br>↓ *Prevotella*<br>▪ **Firmicutes phylum:**<br>↑ *Roseburia*<br>↑ *Oscillospira*<br>↑ *Lachnospiraceae*<br>↑ *Lactobacillus*<br>↑ *Streptococcus*<br>▪ **Verrucomicrobia phylum:**<br>↑ *Akkermansia muciniphila*	• Caesar, R., *et al.* (2015)<br>• Wolters, M. *et al.* (2019)<br>• Millman, J. F. *et al.* (2021)<br>• O'Connor, K. *et al.* (2019)<br>• Brahe, L. K. *et al.* (2015)<br>• Watson, H. *et al.* (2018)<br>• Balfegó, M. *et al.*(2016)<br>• Andersen, A. D., *et al.* (2011)

**Carbohydrates: Refined**	Associated with cognitive impairment	▪ ↑ **Proteobacteria phylum**<br>▪ ↓ **Bacteroidetes phylum**<br>▪ **Verrucomicrobia phylum:**<br>↓ *Akkermansia muciniphila*	• Antonini, M. *et al.* (2019)<br>• Satokari, R. (2020)<br>• Do, M. H. *et al.* (2018)<br>• Shang, W. *et al.* (2017)
**Carbohydrates: Fiber**	Associated with improved cognitive function	▪ **Actinobacteria phylum:**<br>↑ *Bifidobacterium*<br>▪ ↑ **Bacteroidetes phylum:**<br>↑ *Prevotella*<br>▪ **Firmicutes phylum:**<br>↑ *Lactobacillus*<br>↑ *Ruminococcus*<br>↑ *Roseburia*<br>↑ *Eubacterium rectale*	• Halmos, E. P. *et al.* (2015)<br>• Reddy, B. S. *et al.* (1975)<br>• Walker, A. W. *et al.* (2011)<br>• Costabile, A. *et al.* (2008)<br>• Carvalho-Wells, A. L. *et al.* (2010)<br>• Keim, N. L. *et al.* (2014)<br>• Leitch, E. C. M. W. *et al.* (2007)<br>• de Wit, N. *et al.* (2012).<br>• Hildebrandt, M. A. *et al.* (2009)<br>• De Filippo, C. *et al.* (2010)<br>• Lopez-Legarrea, P. *et al.* (2014)
**Protein: Animal-Based**	Associated with cognitive impairment	▪ **Actinobacteria phylum:**<br>↓ *Bifidobacterium adolescentis*<br>▪ **Bacteroidetes phylum:**<br>↑ *Bacteroides*<br>↑ *Alistipes*<br>▪ **Firmicutes phylum:**<br>↑ *Clostridia*<br>▪ **Proteobacteria phylum**<br>↑ *Bilophila*	• David, L. A. *et al.* (2014)<br>• Cotillard, A. *et al.* (2013)<br>• Hentges, D. J. *et al.* (1977)<br>• Russell, W. R. *et al.* (2011)<br>• De Filippo, C. *et al.* (2010)<br>• Reddy, B. S. *et al.* (1975)
**Protein: Plant-Based**	Associated with improved cognitive function	▪ **Actinobacteria phylum:**<br>↑ *Bifidobacterium*<br>▪ **Firmicutes phylum:**<br>↑ *Lactobacillus*<br>↓ *Clostridium perfringens*<br>▪ **Bacteroidetes phylum:**<br>↓ *Bacteroides fragilis*	• Reddy, B. S. *et al.* (1975)<br>• Dominika, Ś. *et al.* (2011)<br>• Romond, M. B. *et al.* (1998)
**Polyphenols**	Associated with improved cognitive function	▪ **Actinobacteria phylum:**<br>↑ *Bifidobacterium*<br>▪ **Firmicutes phylum:**<br>↑ *Lactobacillus*<br>↓ *Staphylococcus aureus*<br>↓ *Clostridium*<br>▪ **Proteobacteria phylum:**<br>↓ *Salmonella typhimurium*	• Queipo-Ortuño, M. I. *et al.* (2012)<br>• Bialonska, D. *et al.* (2010)<br>• Druart, C. *et al.* (2014)<br>• Tzounis, X. *et al.* (2008)<br>• Eid, N. *et al.* (2014)<br>• Cuervo, A. *et al.* (2014)<br>• Vendrame, S. *et al.* (2011)<br>• Jin, J. S., Touyama, M. *et al.* (2012)<br>• Tzounis, X. *et al.* (2011)<br>• Cueva, C. *et al.* (2013)<br>• Parkar, S. G. *et al.* (2008)<br>• Lee, H. C. *et al.* (2006)
**Alcohol: Red wine**	Associated with improved cognitive function	▪ **Bacteroidetes phylum:**<br>↑ *Bacteroides*	• Queipo-Ortuño, M. I. *et al.* (2012)<br>• Nash, V. *et al.* (2018)
**Alcohol: Binge Drinking**	Associated with cognitive impairment	▪ ↑ **Bacteroidetes phylum**<br>▪ ↓ **Firmicutes phylum:**<br>↑ *Enterococci*<br>↑ *Clostridium*<br>↑ *Holdemania*<br>↓ *Faecalibacterium*<br>▪ ↑ **Proteobacteria phylum:**<br>↑ *E. Coli*<br>↑ *Klebsiella*<br>↑ *Pasteurella*<br>↑ *Proteus*<br>↑ *Pseudomonas*	• Yan, A. W. *et al.* (2012)<br>• Mutlu, E. A. *et al.* (2012)<br>• Bjørkhaug, S. T. *et al.* (2019)

		↑ *Shigella*<br>• ↑ *Sutterella*<br>▪ ↑ **Verrucomicrobia phylum**	
